# An ensemble model of QSAR tools for regulatory risk assessment

**DOI:** 10.1186/s13321-016-0164-0

**Published:** 2016-09-22

**Authors:** Prachi Pradeep, Richard J. Povinelli, Shannon White, Stephen J. Merrill

**Affiliations:** 1 National Center for Computational Toxicology (ORISE Fellow), US EPA, Research Triangle Park, NC USA; 2 Electrical and Computer Engineering Department, Marquette University, Milwaukee, WI USA; 3 Department of Mathematics, Statistics, and Computer Science, Marquette University, Milwaukee, WI USA; 4 Lombardi Comprehensive Cancer Center, Georgetown University Medical Center, Washington, DC USA

**Keywords:** Computational toxicology, In silico QSAR tools, Hybrid QSAR models, Ensemble models, Risk assessment

## Abstract

Quantitative structure activity relationships (QSARs) are theoretical models that relate a quantitative measure of chemical structure to a physical property or a biological effect. QSAR predictions can be used for chemical risk assessment for protection of human and environmental health, which makes them interesting to regulators, especially in the absence of experimental data. For compatibility with regulatory use, QSAR models should be transparent, reproducible and optimized to minimize the number of false negatives. In silico QSAR tools are gaining wide acceptance as a faster alternative to otherwise time-consuming clinical and animal testing methods. However, different QSAR tools often make conflicting predictions for a given chemical and may also vary in their predictive performance across different chemical datasets. In a regulatory context, conflicting predictions raise interpretation, validation and adequacy concerns. To address these concerns, ensemble learning techniques in the machine learning paradigm can be used to integrate predictions from multiple tools. By leveraging various underlying QSAR algorithms and training datasets, the resulting consensus prediction should yield better overall predictive ability. We present a novel ensemble QSAR model using Bayesian classification. The model allows for varying a cut-off parameter that allows for a selection in the desirable trade-off between model sensitivity and specificity. The predictive performance of the ensemble model is compared with four in silico tools (Toxtree, Lazar, OECD Toolbox, and Danish QSAR) to predict carcinogenicity for a dataset of air toxins (332 chemicals) and a subset of the gold carcinogenic potency database (480 chemicals). Leave-one-out cross validation results show that the ensemble model achieves the best trade-off between sensitivity and specificity (accuracy: 83.8 % and 80.4 %, and balanced accuracy: 80.6 % and 80.8 %) and highest inter-rater agreement [kappa (*κ*): 0.63 and 0.62] for both the datasets. The ROC curves demonstrate the utility of the cut-off feature in the predictive ability of the ensemble model. This feature provides an additional control to the regulators in grading a chemical based on the severity of the toxic endpoint under study.

**Electronic supplementary material:**

The online version of this article (doi:10.1186/s13321-016-0164-0) contains supplementary material, which is available to authorized users.

## Background

Chemical risk assessment associated with chemical exposure is necessary for the protection of human and environmental health. Toxicity or adverse effects are major reasons for failure of a potential pharmaceutical, an industrial chemical or a medical device [1–3]. Regulatory risk assessment is the process that ensures marketing of safe and effective drugs, medical devices and other consumer products. Regulatory decisions are primarily dependent on the short and long term toxic and clinical effects of chemicals. Conventional methods of risk assessment (in vivo experiments and clinical trials) are performed only after product development, and are expensive and time-consuming. Although in vivo experimental studies are the most accurate method for identifying the toxic effects induced by a xenobiotic, time and cost associated with them for new chemical regulation renders them ineffective for regulatory risk assessment.

In silico approaches to predictive toxicology focus on building quantitative structure activity relationship (QSAR) models that can mimic the results of in vivo studies. In silico methods are appealing because they provide a faster alternative to otherwise time-consuming laboratory and clinical testing methods [4, 5]. Currently, several commercial (free or proprietary) and open source in silico QSAR tools are available that can predict the toxic effects of a chemical based on its chemical structure [6, 7]. QSAR models are widely used for identification of chemicals that have a desired biological effect (*e.g.* drug leads) or for early prediction of potential toxic effects in the pharmaceutical industry. In contrast to industrial use, regulatory use of QSAR models is very different. In a regulatory application, QSAR models can be used to: (1) supplement experimental data, (2) support prioritization in the absence of experimental data, and (3) replace experimental animal testing methods [8, 9].

Several QSAR models have been used and validated by United Sates (US) regulatory agencies and are rapidly gaining impetus in the European Union (EU) too [10–13]. In the EU, the REACH (Registration, Evaluation, Authorization and Restriction of Chemicals) initiative mandates risk assessment of new and existing chemicals [14]. Similar to REACH, the Organization for Economic Co-operation and Development (OECD), also has a set of internationally agreed upon validation principles for regulatory acceptance of QSAR models [15].

In view of the possible uses of QSAR tools, regulators often use predictions from multiple QSAR tool for arriving at a decision. However, different QSAR tools often make conflicting predictions for a given chemical and also vary in their predictive ability for different classes of chemicals. Often, the validation of a particular QSAR tool and sufficient confidence that it can be used reliably for a given chemical is not available, which makes handling conflicting predictions and determining the best prediction difficult [16]. Transparency in predictions is crucial in developing safety assessment decisions and reports, which makes the use of QSAR tools challenging for a regulatory risk assessment. In this manuscript, we present a Bayesian ensemble model of QSAR tools with improved prediction accuracy and reliability. In the following sections, we discuss the present state-of-the-art, describe ensemble methodology, Bayes classification and present a comparative analysis of the Bayes ensemble model.

## Related work

There are studies that investigate methods for combining predictions from multiple QSAR tools to gain better predictive performance for various toxic endpoints: (1) Several QSAR models were developed and compared using different clustering algorithms (multiple linear regression, radial basis function neural network and support vector machines) to develop hybrid models for bioconcentration factor (BCF) prediction [17]; (2) QSAR models implementing cut-off rules were used to determine a reliable and conservative consensus prediction from two models implemented in VEGA [18] for BCF prediction [19]; (3) Predictive performance of four QSAR tools (Derek [20, 21], Leadscope [22], MultiCASE [23] and Toxtree [24]) were evaluated and compared to the standard Ames assay [25] for mutagenicity prediction. Pairwise hybrid models were then developed using AND (accepting positive results when both tools predict a positive) and OR combinations (accepting positive results when either one of the tool predicts a positive) [25–27]; (4) A similar AND/OR approach was implemented for the validation and construction of a hydrid QSAR model using MultiCASE and MDL-QSAR [28] tools for carcinogenicity prediction in rodents [29]. The work was extended using more tools (BioEpisteme [30], Leadscope PDM, and Derek) to construct hybrid models using majority consensus predictions in addition to AND/OR combinations [31].

The results of these studies demonstrate that: (1) None of the QSAR tools perform significantly better than others, and they also differ in their predictive performance based upon the toxic endpoint and the chemical datasets under investigation, (2) Hybrid models have an improved overall predictive performance in comparison to individual QSAR tools, and (3) Consensus-positive predictions from more than one QSAR tool improved the identification of true positives. The underlying idea is that each QSAR model brings a different perspective of the complexity of the modeled biological system and combining them can improve the classification accuracy. However, consensus-positive methods are prone to introducing a conservative nature in discarding a potentially non-toxic chemical based on false positive prediction. Therefore, we propose an ensemble learning approach for combining predictions from multiple QSAR tools that addresses the drawbacks of consensus-positive predictions [32, 33]. Hybrid QSAR models using ensemble approaches have been developed for various biological endpoints like cancer classification and prediction of ADMET properties [34–36] but not for toxic endpoints. In this study, a Bayesian ensemble approach is investigated for carcinogenicity prediction, which is discussed in more details in the next section.

## Methods

### QSAR tools

Four open-source QSAR tools were used to make predictions about carcinogenicity for chemicals used in this study:

1. *OECD ToolBox* Chemicals were screened for two mutagenic alerts: (1) in vitro mutagenicity alerts by ISS (Ames mutagenicity), and (2) in vivo mutagenicity alerts by ISS (Micronucleus assay), and two carcinogenic alerts: (1) carcinogenic (genotoxic and non-genotoxic) alerts by ISS, and (2) oncology primary classifications) profiling alerts. A positive result in a profiling category for any chemical substance was considered a positive carcinogenicity prediction for the test chemical [37].
2. *Danish QSAR* Chemicals were screened in the database for mutagenicity, mutagenicity in vivo, and carcinogenicity. A positive or equivalent prediction in any category was recorded as a positive carcinogenicity prediction for the test chemical [38].
3. *Lazar* Chemicals were queried in the tool using the DSSTox carcinogenic potency DBS multicellcall endpoint and the two available mutagenic endpoints (DSSTox carcinogenic potency DBS mutagenicity and Kazius-Bursi Salmonella mutagenicity). A positive result for either category was recorded as a positive carcinogenicity prediction for the test chemical.
4. *Toxtree* Chemicals were queried in the Toxtree using the Benigni/Bossa Rulebase (for mutagenicity and carcinogenicity). If a potential carcinogenic alert based on any QSAR model or if any structural alert for genotoxic and non-genotoxic carcinogenicity was reported, then the prediction was recorded as a positive carcinogenicity prediction for the test chemical.

### Datasets

Two datasets, that consist of both carcinogenic and non-carcinogenic chemicals, were used for training and testing:

1. *Air toxins* A set of chemicals potentially emitted in the industrial environment was obtained from the Western Australia Department of Health. The dataset consists of 332 chemicals with a carcinogen to non-carcinogen ratio of 114:218.
2. *Gold carcinogenic potency database (CPDB)* The CPDB houses results from chronic, long-term animal cancer tests on a variety of chemicals [39]. The database was screened for all chemicals with positive or negative carcinogenic data in both male and female mice and/or rats. A chemical was considered carcinogenic if the response in either species and/or gender had a TD50 data, else it was considered non-carcinogenic. The final dataset consists of 480 chemicals with a carcinogen to non-carcinogen ratio of 258:222.

Selection of chemicals in each dataset was based on the availability of experimental in vivo carcinogenicity data [obtained from the Carcinogenic Potency Database and Chemical Carcinogenesis Research Information System (CCRIS [40])] and predictions from all four QSAR tools. The list of chemicals in both the datasets is provided in Tables 1 and 2 of the Additional file 1.

### Bayes ensemble model

A Bayes ensemble model is based on the concept of prior probabilities [41, 42]. The model uses training data for classification by estimating uncertain quantities using the Bayes theorem. Bayes theorem uses the training data as evidence (E) for a seen outcome (O), to construct a probability for predicting the outcome when the evidence is seen in the future [43]. The probability of observing the outcome in the past (training dataset) is termed as the prior probability (*P*(*O*)) and the probability of predicting the outcome occuring in the future is termed as the posterior probability ($P(O\vert E)$). The Bayes theorem calculates the posterior probability using Eq. (1).

1 $$\begin{aligned} P(O|E)=\frac{P(E|O)P(O)}{P(E)}, \end{aligned}$$

where *P*(*O*) is the probability of the outcome and *P*(*E*) is the probability of the evidence. In a binary classification problem, the final predicted class is the one with a higher value of $P(O\vert E)$.

In this study, the training data consisted of predictions from four QSAR tools and true experimental class about the nature of the chemical (carcinogenic or non-carcinogenic). Each tool was used to make a prediction about the class ($\omega$), which is recorded as 1 or 0 representing carcinogenic and non-carcinogenic, respectively. Since there were four QSAR tools, the possible number of combinations of predictions is $k= 2^{4}\,(=16)$. Each unique prediction combination is represented by the vector $s_{k}$. The posterior probability of a chemical being carcinogenic ($\omega =1$) or non-carcinogenic ($\omega =0$) associated with each prediction combination, $P(\omega \vert s=s_{k})$ is then calculated using Eq. (2).

2 $$\begin{aligned} P(\omega |s=s_{k})_{k}&= \frac{P(s_{k}|\omega )P(\omega )}{P(s_{k})}, \end{aligned}$$

where, $s_{k}$ is the prediction combination for the test chemical, $P(s_{k} \vert \omega )$ is the prior probability of observing a prediction combination $s_{k}$ given that a chemical is carcinogenic or non-carcinogenic, $P(\omega )$ is the probability of a chemical being carcinogenic or non-carcinogenic and $P(s_{k})$ is the probability of a particular prediction combination from the QSAR tools. So, for each prediction combination ($s_{k}$) there is an associated posterior probability ($P(\omega |s=s_{k})$), which is used to make the final classification ($\omega '$). The algorithm was implemented in Matlab R2015a [44] and the source code is provided in the Additional file 2.

### Algorithm

The approach outlined above is implemented in two steps for estimation of the final classification ($\omega '$):

Step 1.: The posterior probability of a test chemical being carcinogenic was calculated from Eq. (3) and was used to construct a decision table as shown in Table 1 for both datasets.
  3 $$\begin{aligned} P(\omega =1|s=s_{k}) = \frac{P(s_{k}|\omega =1)P(\omega =1)}{P(s_{k})} \end{aligned}$$
  where,
  4 $$\begin{aligned} P(s_{k}|\omega =1)&=\frac{N_{(\omega =1, s_{k})}}{N_{(\omega =1)}}, \end{aligned}$$
  5 $$\begin{aligned} P(\omega =1)&=\frac{N_{(\omega =1)}}{N}, \;and \end{aligned}$$
  6 $$\begin{aligned} P(s_{k})&=\frac{N_{s_{k}}}{N}. \end{aligned}$$
  So,
  7 $$\begin{aligned} P(\omega =1|s=s_{k})&= \frac{\left(\frac{N_{(\omega =1, s_{k})}}{N_{(\omega =1)}}\right)\left(\frac{N_{(\omega =1)}}{N}\right)}{\left(\frac{N_{s_{k}}}{N}\right)}\end{aligned}$$
  8 $$\begin{aligned}&=\frac{N_{(\omega =1, s_{k})}}{N_{s_{k}}}, \end{aligned}$$
  where $N_{s_{k}}$ was the number of chemicals with a prediction combination $s_{k}$ in the training dataset, $N_{(\omega =1)}$ was the total number of carcinogens in the training dataset, $N_{(\omega =1, s_{k})}$ was the number of carcinogens with prediction combination $s_{k}$, *N* was the total number of chemicals in the training dataset and *k* ranges from 1 to 16. Tables 3 and 4 in the Additional file 1 list the number of samples in each of the 16 prediction classes for both datasets.

**Table 1:** Prediction combination table with posterior probability, $P(\omega |s=s_{k})$, for each combination number, $s_{k}$, which represents a prediction combination from each of the four QSAR tools

Combination number	Tool 1	Tool 2	Tool 3	Tool 4	Posterior probability
$s_{1}$	0	0	0	0	$P(\omega |s=s_{1})$
$s_{2}$	0	0	0	1	$P(\omega |s=s_{2})$
$s_{3}$	0	0	1	0	$P(\omega |s=s_{3})$
$s_{4}$	0	0	1	1	$P(\omega |s=s_{4})$
$s_{5}$	0	1	0	0	$P(\omega |s=s_{5})$
$s_{6}$	0	1	0	1	$P(\omega |s=s_{6})$
$s_{7}$	0	1	1	0	$P(\omega |s=s_{7})$
$s_{8}$	0	1	0	1	$P(\omega |s=s_{8})$
$s_{9}$	0	1	1	1	$P(\omega |s=s_{9})$
$s_{10}$	1	0	0	0	$P(\omega |s=s_{10})$
$s_{11}$	1	0	0	1	$P(\omega |s=s_{11})$
$s_{12}$	1	0	1	0	$P(\omega |s=s_{12})$
$s_{13}$	1	0	1	1	$P(\omega |s=s_{13})$
$s_{14}$	1	1	0	0	$P(\omega |s=s_{14})$
$s_{15}$	1	1	0	1	$P(\omega |s=s_{15})$
$s_{16}$	1	1	1	1	$P(\omega |s=s_{16})$

**Fig. 1:**
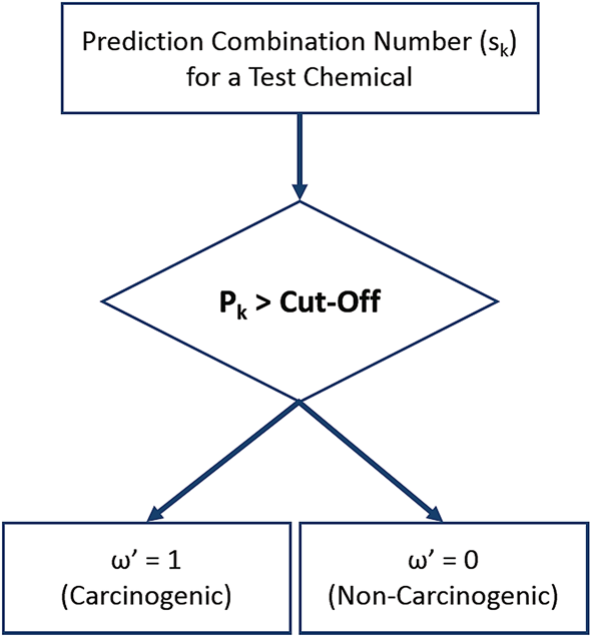
Bayesian classifier ensemble for predicting carcinogenicity. The posterior probability, $P_{k}$, as determined from Table 1 is compared with a variable cut-off between 0 and 1

Step 2.: For a new test chemical, the prediction combination vector $s_{k}$ was determined and was used to look up the posterior probability $P(\omega =1|s=s_{k})$ or $P_{k}$ associated with it from the decision table. The final prediction $(\omega ')$ was estimated based on the value of $P_{k}$, which was compared to a variable *cut-off* as outlined in Fig. 1. The cut-off represents the value of posterior probability beyond which a new test chemical can be classified as carconogenic. The value of the cut-off can be varied (between 0 and 1) leading to different decision points for the final classification.

The Bayes ensemble model is, thus, very powerful in giving a user the flexibility of adjusting the cut-off to reach a desired level of sensitivity and specificity as demonstrated in the results. The flexibility in changing the cut-off also makes the model endpoint independent.

### Model validation

One of the major concerns with the use of QSAR tools for a regulatory purpose is the reliability in their predictions. QSAR tools need to be assessed for their scientific validity so that regulatory organizations have a sound scientific basis for decision making. The OECD member countries agreed upon a set of principles as guidelines for scientifically validating a QSAR model. In accordance with these guidelines, external model validation was performed and a range of model statistics were calculated for a comprehensive performance analysis. The leave one out cross validation (LOOCV) technique was used for external validation where *N * models were developed each with $(N-1)$ chemicals as training set and 1 chemical as the test set. The following standard metrics were then calculated to assess the performance of the models:

9 $$\begin{aligned} Accuracy=\frac{TP+TN}{TP+FN+TN+FP}, \end{aligned}$$

10 $$\begin{aligned} Sensitivity\; (SN)=\frac{TP}{TP+FN}, \end{aligned}$$

11 $$\begin{aligned} Specificity \; (SP)=\frac{TN}{TN+FP}, \end{aligned}$$

12 $$\begin{aligned} Balanced\;Accuracy \;(BA) =\frac{SP + SN}{2} \end{aligned}$$

13 $$\begin{aligned} PPV=\frac{TP}{TP+FP}, \; and \end{aligned}$$

14 $$\begin{aligned} NPV=\frac{TN}{TN+FN}, \end{aligned}$$

where TP is the number of true positives, TN is the number true negatives, FP is the number of false positives, and FN is the number of false negatives reported in the tests. Accuracy or concordance is a measure of correctness of overall predictions. Sensitivity is a measure of correctness in prediction of positives or toxic chemicals and specificity is a measure of correctness in prediction of negatives or non-toxic chemicals. Balanced accuracy (BA) is the arithmetic mean of sensitivity and specificity and represents a trade-off between the two values. Positive predictive value (PPV) is the proportion of positives or toxic chemicals that are correctly predicted and negative predictive value (NPV) is the proportion of negatives or non-toxic chemicals that are correctly predicted. High sensitivity or low false negatives is especially important under REACH requirements. BA, PPV and NPV are crucial in understanding the predictive power of the models based on the representation of carcinogenic and non-carcinogenic chemicals in the training datasets.

The OECD guidelines emphasize appropriate measures of goodness-of-fit, robustness and predictivity of QSAR models. Several reports discuss potential techniques for internal and external measures of model validation [45–47]. Therefore, in addition to the standard metrics two conceptually simpler statistical parameters are also calculated, which are indicative of overall concordance and performance of each model as compared to chance and each other:

1. *Cohen’s Kappa (κ)* The Kappa coefficient is a measure of pairwise inter-rater agreement or specific agreement compared to a chance agreement. It is calculated as below:
  15 $$\begin{aligned}\kappa =\frac{(TP+TN)-\left(\frac{(TP+FN)(TP+FP)+(FP+TN)(FN+TN)}{N}\right)} {1-\left(\frac{(TP+FN)(TP+FP)+(FP+TN)(FN+TN)}{N}\right)}. \end{aligned}$$
  In this study, the Kappa coefficient is used to compare how well the predictions from various tools agree with the experimental or true values. Values of $\kappa =0$, $0.41< \kappa <0.60$, $0.61< \kappa < 0.80$ and $\kappa =1$ represent no, moderate, substantial and perfect agreement, respectively [48, 49].
2. *Receiver Operating Characteristics (ROC) Curve* A ROC curve is a plot of true positive rate (sensitivity) and the false positive rate (1-specificity). A ROC curve demonstrates how the performance of a binary classifier changes as the threshold parameters are varied [50]. Area under the ROC curve can be used to compare the classification tools; higher area implies a better classification.

## Results and discussion

### Accuracy, sensitivity, specificity, balanced accuracy, PPV and NPV

Statistical performance of the ensemble model in comparison to the various QSAR tools is summarized in Tables 2 and  3. The statistics for the Bayes ensemble model are presented for three different cut-offs, which demonstrate the utility of the cut-off feature. As shown, the accuracy (>80 %), balanced accuracy (>78 %), PPV (>79 %) and NPV (>79 %) of the Bayes ensemble model is highly improved compared to the base classifiers (QSAR tools) for both the datasets. The specificity was substantially improved which adheres with the REACH legislatives emphasis on the reduction of false negatives.

**Table 2:** Performance metrics for air toxins dataset

Model	Accuracy (%)	SN (%)	SP (%)	BA (%)	PPV (%)	NPV (%)	Kappa ($\kappa$)
Toxtree	75.56	68.18	79.51	73.85	64.10	82.32	0.47
Lazar	75.24	74.55	75.61	75.08	62.12	84.70	0.48
Danish QSAR	74.29	80.91	70.73	75.82	59.73	87.35	0.48
OECD toolbox	76.19	69.09	80.00	74.55	64.96	82.83	0.48
Bayes ensemble (Cut-off = 0.4)	83.81	70.00	91.22	80.61	81.05	85.00	0.63
Bayes ensemble (Cut-off = 0.5)	83.81	70.00	91.22	80.61	81.05	85.00	0.63
Bayes ensemble (Cut-off = 0.6)	82.22	65.45	91.22	78.34	80.00	83.11	0.59

**Table 3:** Performance metrics for the CPDB dataset

Model	Accuracy (%)	SN (%)	SP (%)	BA (%)	PPV (%)	NPV (%)	Kappa ($\kappa$)
Toxtree	66.04	84.50	44.59	64.55	63.93	71.22	0.30
Lazar	80.63	86.05	74.32	80.19	79.57	82.09	0.61
Danish QSAR	65.00	91.09	34.68	62.89	61.84	77.00	0.27
OECD toolbox	64.79	84.50	41.89	63.20	62.82	69.93	0.27
Bayes ensemble (Cut-off = 0.4)	81.04	83.33	75.23	79.28	80.14	82.27	0.62
Bayes ensemble (Cut-off = 0.5)	80.21	84.50	75.23	79.87	79.85	80.68	0.60
Bayes ensemble (Cut-off = 0.6)	80.42	84.50	77.03	80.77	80.83	79.91	0.61

The statistics demonstrate the inability of any particular QSAR tool to make consistent predictions across different chemical datasets. In case of Bayes ensemble model, varying the cut-off leads to perfect sensitivity (cut-off = 0) or perfect specificity (cut-off = 1). However, cut-off values of 0.4, 0.5 and 0.6 result in a balanced sensitivity and specificity with only a minor change in all calculated statistics for both the datasets. This demonstrates the robustness of the Bayes ensemble model. Additionally, pairwise Student’s t-tests was used to establish statistically significant differences between the predictions from the Bayes ensemble model (cut-off = 0.5) and all the tools at a 5 % significance level, for both datasets.

### Cohen’s Kappa coefficient

As seen in Tables 2 and 3, the Bayes ensemble model has the highest Kappa coefficient. This means that the Bayes ensemble predictions best concur with the experimental data. Toxtree, Danish QSAR and the OECD Toolbox demostrate less than moderate agreement with the experimental values for both the datasets. The Bayes ensemble model with cut-off $=0.4$ has a $\kappa > 0.62$ for both the datasets. It is an indication of stronger and more substantial agreement with the experimental values compared to the other QSAR tools.

### ROC curve

**Fig. 2:**
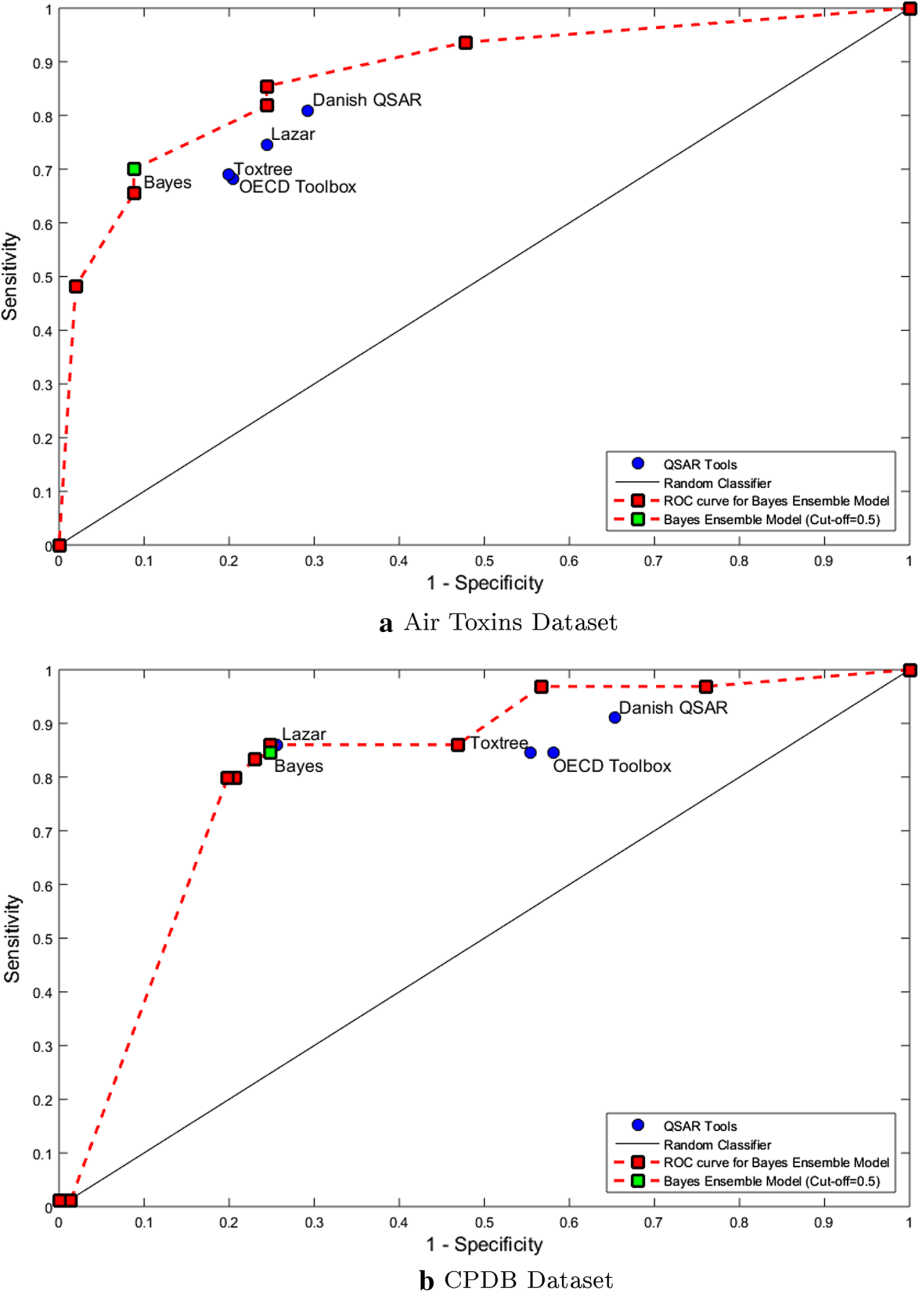
Receiver operator characteristics (ROC) curve of Bayes ensemble model as compared to other QSAR tools. The Bayes model at different thresholds is depicted by *red points*, at 0.5 cut-off by *green point* and the base QSAR tools by *blue points*. The ROC plot for the Bayes ensemble model is depicted by the *red dotted line*. **a** Air toxins dataset, **b** CPDB dataset

Figure 2 shows the receiver operating characteristics plot for all the QSAR tools and the Bayes ensemble model. An ideal binary predictor would have zero false predictions and so the desired point on the ROC curve is top left corner where sensitivity is one and (1-specificity) is zero. The black line corresponds to the performance of a random classifier, which does not have any preferences in binary outcomes. The higher the area under the ROC curve, the greater is the predictive ability of the model. The tools give a binary prediction, therefore, they are represented as a point on the ROC plot. In the case of Bayes ensemble model, a curve can be traced for each sensitivity-specificity combination obtained after changing the value of the cut-off. In this study, the cut-off is varied between 0 and 1 with a step size of 0.1 allowing for 11 decision points for model validation. Hence, the ROC plot consists of data points corresponding to each value of cut-off, which can be traced to obtain a ROC curve. The ROC curve for the Bayes ensemble model is higher than all the other tools implying better quality of predictions.

*The variable cut-off in the ROC curve can be adjusted to select a trade-off between sensitivity and specificity. This feature provides an additional control to the regulating agencies in grading a chemical based on the severity of the toxic endpoint under study*. It exhibits user-control and flexibility in the predictive ability of the ensemble model.

Overall, the results show that the Bayes ensemble model is better and more consistent with respect to different in silico QSAR tools. The model combines predictions from various in silico tools in a transparent and reproducible manner. It can also be optimized to reduce the number of false predictions while maintaining flexibility in addressing other considerations in making these predictions.

## Conclusion

The results of this study demonstrate that different QSAR tools vary in the quality of predictions depending on the underlying algorithm and training datasets. Ensemble machine learning presents a new approach for combining the predictions from multiple QSAR tools. The strength of an ensemble model depends on the diversity of the algorithm and the predictive ability of the base models. Each individual tool has its strengths and weaknesses and an ensemble model enables leveraging the benefits of individual tools, minimizing the impact of their algorithmic differences and increase in chemical space coverage.

The Bayes ensemble model presented here is consistent in its performance across both the datasets. The results specifically show improved (1) accuracy and balanced accuracy in the predictions, (2) specificity and positive predictive value, which are an indication of reduction in false positive predictions, and (3) Kappa coefficient, across both the datasets. The statistics demonstrate how ensemble machine learning methods can be used to increase the capability of consensus QSAR models for toxicity prediction.

The Bayes ensemble model offers flexibility in achieving a desired trade-off between sensitivity and specificity. It also demonstrares how multiple QSAR tools with different complexity and accuracy can be used together for developing more reliable predictors. The results suggest that ensemble modeling techniques are a good strategy for refining hybrid models and to tailor their use based on the severity and concerns associated with the toxic endpoint under study. We presented an example application with Toxtree, Lazar, OECD Toolbox, and Danish QSAR, and two different classes of chemical datasets for carcinogenicity prediction. This approach can be extended to different tools and different kinds and sizes of chemical datasets for different toxic endpoints as well.

## Additional files

10.1186/s13321-016-0164-0 Dataset 1: Air Toxins. **Section S2.** Dataset 2: Subset of Carcinogenic Potency Database. **Section S3.** Distribution of prediction combinations.
10.1186/s13321-016-0164-0 Matlab generated code file and is self-explanatory.
