# Supplementary material for: An ensemble model of QSAR tools for regulatory risk assessment
Source: J Cheminform. 2016 Sep 22;8:48. doi: 10.1186/s13321-016-0164-0 (PMC5034616; doi:10.1186/s13321-016-0164-0)
Supplement: Supplementary file 1 — 10.1186/s13321-016-0164-0 Dataset 1: Air Toxins. Section S2. Dataset 2: Subset of Carcinogenic Potency Database. Section S3. Distribution of prediction combinations. [file 13321_2016_164_MOESM1_ESM.pdf]

S1 Dataset 1: Air Toxins

Table S1: List of Chemicals for Dataset 1 (Air Toxins)

No.	CASRN	Chemical Name
1	2278-53-7	[R-(E)]-5-isopropyl-8-methylnona-6,8,-dien-2-one
2	7287-82-3	1-(2-methylphenyl)ethanol
3	630-20-6	1,1,1,2-tetrachloroethane
4	71-55-6	1,1,1-Trichloroethane
5	79-00-5	1,1,2-trichloroethane
6	75-34-3	1,1-dichloroethane
7	75-35-4	1,1,-Dichloroethylene (1,1-DCE)
8	156-59-2/156-60-5	1,2 (trans)-dichloroethylene
9	87-61-6	1,2,3-trichlorobenzene
10	96-18-4	1,2,3-Trichloropropane
11	95-94-3	1,2,4,5-tetrachlorobenzene
12	120-82-1	1,2,4-trichlorobenzene
13	95-63-6	1,2,4-trimethylbenzene
14	930-87-0	1,2,5-trimethylpyrrole
15	84-78-6	1,2-benzenedicarboxylic acid, butyl octy
16	96-12-8	1,2-dibromo-3-chloropropane
17	106-93-4	1,2-Dibromoethane
18	95-50-1	1,2-dichlorobenzene
19	107-06-2	1,2-dichloroethane
20	78-87-5	1,2-Dichloropropane
21	122-66-7	1,2-diphenylhydrazine
22	106-88-7	1,2-Epoxybutane (EBU)
23	2235-12-3	1,3,5 hexatriene
24	108-67-8	1,3,5-trimethylbenzene
25	99-35-4	1,3,5-trinitrobenzene
26	106-99-0	1,3-Butadiene
27	542-92-7	1,3-cyclopentadiene
28	541-73-1	1,3-dichlorobenzene
29	542-75-6	1,3-Dichloropropene
30	99-65-0	1,3-dinitrobenzene
31	646-06-0	1,3-dioxalane
32	106-46-7	1,4-Dichlorobenzene
33	123-91-1	1,4-dioxane
34	575-43-9	1,6-dimethylnaphthalenea
35	822-06-0	1,6-Hexamethylene disocyanate
36	75-68-3	1-Chloro-1,1-difluoroethane
37	622-96-8	1-ethenyl-4-methyl-benzene
38	621-32-9	1-ethoxy-3-methyl-benzene

No.	CASRN	Chemical Name
39	874-41-9	1-ethyl-2,4-dimethyl-benzene
40	620-14-4	1-ethyl-3-methyl-benzene
41	622-96-8	1-ethyl-4-methylbenzene
42	592-41-6	1-hexene
43	3034-50-2	1H-imidazole-4-carbaldehyde
44	99-87-6	1-isopropyl-4-methylbenzene
45	2886-59-1	1-methoxy-1,4-cyclohexadiene
46	767-59-9	1-methyl-1H-indene
47	99-85-4	1-methyl-4-(1-methylethyl)1,4- cyclohexadiene
48	3333-13-9	1-methyl-4-(2-propenyl)-benzene
49	90-12-0	1-methylnaphthalene
50	110-66-7	1-pentanethiol
51	103-65-1	1-propylbenzene
52	2409-55-4	2-(1,1-dimethylethyl)-4-methyl-phenol
53	2219-82-1	2-(1,1-dimethylethyl)-6-methyl-phenol
54	4901-51-3	2,3,4,5-tetrachlorophenol
55	58-90-2	2,3,4,6-tetrachlorophenol
56	28790-86-5	2,3,4-trimethyl-2-cyclopenten-1-one
57	431-03-8	2,3-butanedione
58	83-33-0	2,3-dihydro-1H-inden-1-one
59	526-75-0	2,3-dimethyl-phenol
60	118-96-7	2,4,6-trinitrotoluene
61	96-76-4	2,4-bis(1,1-dimethylethyl)-phenol
62	120-83-2	2,4-dichlorophenol
63	13494-06-9	2,4-dimethyl-1,3-cyclopentanedione
64	565-80-0	2,4-dimethyl-3-pentanone
65	105-67-9	2,4-dimethylphenol
66	51-28-5	2,4-dinitrophenol
67	26471-62-5	2,4/2,6-Toluene diisocyanate mixture (TDI)
68	5875-45-6	2,5-bis(1,1-dimethylethyl)-phenol
69	120-52-5	2,5-cyclohexadiene-1,4-dione, bis(O-benzoyloxime)
70	3891-98-3	2,6,10-trimethyldodecane
71	112-35-6	2-[2-(2-methoxyethoxy)ethoxy]-ethanol
72	78-92-2	2-butanol
73	78-93-3	2-butanone
74	532-27-4	2-Chloroacetophenone
75	91-58-7	2-chloronaphthalenea
76	95-57-8	2-chlorophenol
77	693-54-9	2-decanone
78	769-25-5	2-ethenyl-1,3,5-trimethyl-benzene
79	110-80-5	2-Ethoxyethanol

No.	CASRN	Chemical Name
80	1758-88-9	2-ethyl-1,4-dimethylbenzene
81	104-76-7	2-ethyl-1-hexanol
82	1551-06-0	2-ethyl-1H-pyrrole
83	123-05-7	2-ethylhexanal
84	591-78-6	2-Hexanone
85	90-02-8	2-hydroxybenzaldehyde
86	1195-09-1	2-methoxy-5-methylphenol
87	109-86-4	2-Methoxyethanol
88	636-41-9	2-methyl-1H-pyrrole
89	75-66-1	2-methyl-2-propanethiol
90	565-69-5	2-methyl-3-pentanone
91	78-78-4	2-methylbutane
92	91-57-6	2-methylnaphthalenea
93	95-48-7	2-methylphenol
94	75-66-1	2-methyl-propane-2-thiol
95	78-84-2	2-methylpropanal
96	554-14-3	2-methylthiophene
97	7045-71-8	2-methylundecane
98	88-74-4	2-nitroaniline
99	79-46-9	2-Nitropropane
100	821-55-6	2-nonanone
101	111-13-7	2-octanone
102	2809-67-8	2-octyne
103	107-87-9	2-pentanone
104	21915-53-7	2-phenyl-oxiranemethanol
105	75-33-2	2-propanethiol
106	873-94-9	3,3,5-trimethylcyclohexanone
107	119-90-4	3,3-dimethoxybenzidine
108	27129-87-9	3,5-dimethyl-benzenemethanol
109	108-68-9	3,5-dimethyl-phenol
110	26472-00-4	"3a,4,7,7a-tetrahydrodimethyl-4,7-methano-1H-inde"
111	21835-01-8	3-ethyl-2-hydroxy-2-cyclopenten-1-one
112	767-60-2	3-methyl-1H-indene
113	563-80-4	3-methyl-2-butanone
114	590-86-3	3-methylbutanal
115	96-14-0	3-methylpentane
116	108-39-4	3-methylphenol
117	99-09-2	3-nitroaniline
118	24851-98-7	3-oxo-2-pentyl-cyclopentaneacetic acid
119	96-22-0	3-pentanone
120	101-55-3	4-bromophenyl-phenylether

No.	CASRN	Chemical Name
121	59-50-7	4-chloro-3-methylphenol
122	7005-72-3	4-chlorophenyl-phenylether
123	2896-60-8	4-ethyl-1,3-benzenediol
124	4748-78-1	4-ethylbenzaldehyde
125	121-33-5	4-hydroxy-3-methoxybenzaldehyde
126	150-76-5	4-methoxyphenol
127	108-10-1	4-methyl-2-pentanone
128	141-79-7	4-methyl-3-penten-2-one
129	104-87-0	4-methylbenzaldehyde
130	589-18-4	4-methyl-benzenemethanol
131	106-44-5	4-methylphenol(p-cresol)
132	100-01-6	4-nitroaniline
133	3775-01-7	5-benzylidenehydantoin
134	15356-70-4	5-methyl-2-(1-methylethyl)-cyclohexanol
135	17312-76-4	6,6-dimethylundecane
136	514-10-3	abietic acid
137	75-07-0	Acetaldehyde
138	75-05-8	Acetonitrile
139	98-86-2	acetophenone
140	107-02-8	Acrolein
141	79-06-1	Acrylamide
142	79-10-7	Acrylic acid
143	107-13-1	Acrylonitrile
144	107-05-1	Allyl chloride
145	319-84-6	alpha-hexachlorocyclohexane
146	62-53-3	Aniline
147	120-12-7	anthracenea
148	12674-11-2	aroclor 1016
149	100-52-7	benzaldehyde
150	71-43-2	Benzene
151	60-12-8	benzeneethanol
152	56-55-3	benzo(a)anthracenea
153	50-32-8	benzo(a)pyrene
154	191-24-2	benzo(ghi)perylenea
155	65-85-0	benzoic acid
156	100-47-0	benzonitrile
157	100-51-6	benzyl alcohol
158	100-44-7	benzyl chloride
159	319-85-7	beta-hexachlorocyclohexane
160	92-52-4	biphenyl
161	111-44-4	bis(2-chlorethyl)ether

No.	CASRN	Chemical Name
162	108-60-1	bis-1,2-chloroisopropyl ether
163	464-41-5	bornyl chloride
164	108-86-1	Bromobenzene
165	75-27-4	bromodichloromethane
166	75-25-2	bromoform
167	74-83-9	Bromomethane
168	123-72-8	butanal
169	106-97-8	butane
170	107-92-6	butanoic acid
171	128-37-0	butylated hydroxytoluene
172	123-72-8	butyraldehyde
173	56-23-5	Carbon tetrachloride
174	108-90-7	chlorobenzene
175	75-45-6	Chlorodifluoromethane
176	75-00-3	chloroethane
177	218-01-9	chrysene a
178	156-59-2	cis-1,2-dichloroethylene
179	123-73-9/15798-64-8	crotonaldehyde
180	98-82-8	Cumene
181	592-57-4	cyclohexa-1,3-diene
182	110-82-7	Cyclohexane
183	108-94-1	cyclohexanone
184	108-91-8	cyclohexylamine
185	542-92-7	cyclopentadiene
186	124-18-5	decane
187	53-70-3	dibenzo(ah)anthracene a
188	124-48-1	dibromochloromethane
189	75-09-2	Dichloromethane
190	75-71-8	dichlorodifluoromethane
191	62-73-7	Dichlorvos
192	110-81-6	diethyl disulfide
193	84-66-2	diethyl phthalate
194	352-93-2	diethyl sulfide
195	108-83-8	diisobutylketone
196	624-92-0	dimethyl disulfide
197	131-11-3	dimethyl phthalate
198	75-18-3	dimethyl sulfide
199	3658-80-8	dimethyl trisulfide
200	127-19-5	dimethylacetamide
201	2432-89-5	di-n-decyl sebacate
202	117-84-0	di-n-octylphthalate

No.	CASRN	Chemical Name
203	112-40-3	dodecane
204	112-95-8	eicosane
205	506-30-9	eicosanoic acid
206	106-89-8	Epichlorohydrin
207	75-08-1	ethanethiol
208	64-17-5	ethanol
209	75-00-3	Ethyl Chloride
210	111-76-2	Ethylene glycol monobutyl ether (EGBE) (2-Butoxyethanol)
211	97-63-2	ethyl methacrylate
212	62-50-0	ethyl methanesulfonate
213	624-89-5	ethyl methyl sulfide
214	100-41-4	Ethylbenzene
215	106-93-4	ethylene dibromide
216	75-21-8	ethylene oxide
217	64-18-6	formic acid
218	629-78-7	heptadecane
219	142-82-5	heptane
220	111-14-8	heptanoic acid
221	87-68-3	hexachloro-1,3-butadiene
222	118-74-1	hexachlorobenzene
223	77-47-4	Hexachlorocyclopentadiene (HCCPD)
224	67-72-1	Hexachloroethane
225	70-30-4	hexachlorophene
226	544-76-3	hexadecane
227	66-25-1	hexaldehyde
228	110-54-3	n-Hexane
229	95-13-6	indene
230	79-77-6	ionone
231	75-28-5	iso-butane
232	78-59-1	isophorone
233	67-63-0	iso-propanol
234	98-82-8	isopropylbenzene
235	50-21-5	lactic acid
236	97-67-6	malic acid
237	78-85-3	methacrolein
238	126-98-7	methacrylonitrile
239	74-93-1	methanethiol
240	79-20-9	methyl acetate
241	74-83-9	methyl bromide
242	74-87-3	Methyl chloride
243	78-93-3	Methyl ethyl ketone (MEK)

No.	CASRN	Chemical Name
244	108-10-1	Methyl isobutyl ketone (MIBK)
245	80-62-6	Methyl methacrylate
246	298-00-0	methyl parathion
247	1634-04-4	Methyl tert-butyl ether (MTBE)
248	108-87-2	methylcyclohexane
249	96-37-7	methylcyclopentane
250	74-95-3	methylene bromide
251	75-09-2	methylene chloride
252	101-68-8, 9016-87-9	Methylene Diphenyl Diisocyanate and polymeric MDI
253	78-98-8	methylglyoxal
254	91-20-3	Napthalene
255	68-12-2	N,N-Dimethylformamide
256	629-97-0	n-docosane
257	463-82-1	neo-pentane
258	629-94-7	n-heneicosane
259	57-10-3	n-hexadecanoic acid
260	98-95-3	Nitrobenzene
261	924-16-3	n-nitroso-di-n-butylamine
262	86-30-6	n-nitrosodiphenylamine
263	621-64-7	n-nitrosodipropylamine
264	111-84-2	nonane
265	103-65-1	n-propylbenzene
266	14167-59-0	n-tetratriacontane
267	638-68-6	n-triacontane
268	57-11-4	octadecanoic acid
269	111-65-9	octane
270	95-53-4	o-toluidine
271	144-62-7	oxalic acid
272	106-47-8	p-chloroaniline
273	608-93-5	pentachlorobenzene
274	82-68-8	pentachloronitrobenzene
275	87-86-5	pentachlorophenol
276	109-66-0	pentane
277	4292-92-6	pentylcyclohexane
278	85-01-8	phenanthrenea
279	108-95-2	phenol
280	60-12-8	phenylethanol
281	75-44-5	Phosgene
282	85-44-9	phthalic anhydride
283	123-38-6	propanal
284	74-98-6	propane

No.	CASRN	Chemical Name
285	75-33-2	propane-2-thiol
286	115-07-1	propene
287	123-38-6	Propionaldehyde
288	107-98-2	Propylene glycol monomethyl ether (PGME)
289	75-56-9	Propylene oxide
290	129-00-0	pyrenea
291	110-86-1	pyridine
292	3232-37-9	salicylidene benzhydrazide
293	100-42-5	Styrene
294	127-18-4	Tetrachloroethylene
295	629-59-4	tetradecane
296	109-99-9	Tetrahydrofuran
297	7098-22-8	tetratetracontane
298	108-88-3	Toluene
299	79-01-6	Trichloroethylene
300	75-69-4	trichlorofluoromethane
301	67-66-3	trichloromethane
302	629-50-5	tridecane
303	121-44-8	Triethylamine
304	75-50-3	trimethylamine
305	540-84-1	2,2,4-Trimethylpentane
306	791-28-6	triphenylphosphine oxide
307	1120-21-4	undecane
308	110-62-3	valeraldehyde
309	121-33-5	vanillin
310	108-05-4	Vinyl acetate
311	593-60-2	Vinyl bromide
312	75-01-4	Vinyl chloride
313	95-47-6	o-Xylene
314	106-42-3	p-Xylene
315	544-25-2	1,3,5-cycloheptatriene

S2 Dataset 2: Subset of Carcinogenic Potency Database

Table S2: List of Chemicals for Dataset 2 (CPDB)

No.	CASRN	Chemical Name
1	62-73-7	Dichlorvos
2	126-72-7	Tris(2,3-dibromopropyl) phosphate
3	597-25-1	Dimethyl morpholinophosphoramidate
4	52-68-6	Trichlorfon
5	531-18-0	Hexamethylmelamine
6	513-37-1	Dimethylvinyl chloride (DMVC)
7	593-60-2	Vinyl bromide
8	75-02-5	Ethene, fluoro-
9	75-01-4	Ethene, chloro-
10	305-03-3	Chlorambucil
11	50-18-0	Cyclophosphamide
12	148-82-3	Melphalan
13	3546-10-9	Phenesterin
14	51-75-2	Nitrogen mustard
15	3068-88-0	beta-Butyrolactone
16	1955-45-9	Pivalolactone
17	1120-71-4	Propane sultone
18	57-57-8	Propiolactone
19	106-92-3	Allyl glycidyl ether
20	101-90-6	Diglycidyl resorcinol ether, technical grade
21	77-83-8	Ethyl-3-methyl-3-phenylglycidate
22	75-21-8	Ethylene oxide
23	106-87-6	4-Vinyl-1-cyclohexene diepoxide
24	556-52-5	Glycidol
25	57-39-6	Metepa
26	122-60-1	Phenyl glycidyl ether
27	75-56-9	1,2-Propylene oxide
28	96-09-3	Styrene oxide
29	52-24-4	Tris(aziridinyl)-phosphine sulfide (thio-tepa)
30	298-18-0	1,2,3,4-Diepoxybutane DL
31	106-88-7	1,2-Epoxybutane
32	100-44-7	Benzyl chloride
33	3296-90-0	2,2-Bis(bromomethyl)-1,3-propanediol, technical grade
34	108-60-1	Bis(2-chloro-1-methylethyl)ether, technical grade
35	75-27-4	Bromodichloromethane
36	109-69-3	n-Butyl chloride
37	75-88-7	2-Chloro-1,1,1-trifluoroethane
38	532-27-4	2-Chloroacetophenone (CN)

No.	CASRN	Chemical Name
39	124-48-1	Chlorodibromomethane
40	107-30-2	Chloromethyl methyl ether
41	96-12-8	1,2-Dibromo-3-chloropropane
42	106-93-4	1,2-Dibromoethane
43	107-06-2	1,2-Dichloroethane
44	78-87-5	1,2-Dichloropropane (propylene dichloride)
45	72-56-0	Di(p-ethylphenyl)dichloroethane
46	306-83-2	Ethane, 2,2-dichloro-1,1,1-trifluoro-
47	144-48-9	Iodoacetamide
48	75-47-8	Iodoform
49	3778-73-2	Isophosphamide
50	576-68-1	Mannitol nitrogen mustard
51	74-83-9	Methyl bromide
52	79-11-8	Monochloroacetic acid
53	79-34-5	1,1,2,2-Tetrachloroethane
54	15318-45-3	Thiamphenicol
55	75-25-2	Tribromomethane
56	79-00-5	1,1,2-Trichloroethane
57	96-18-4	1,2,3-Trichloropropane
58	542-88-1	Bis(chloromethyl) ether
59	74-96-4	Bromoethane (ethyl bromide)
60	75-45-6	Methane, chlorodifluoro-
61	75-00-3	Chloroethane
62	593-70-4	Chlorofluoromethane
63	75-34-3	1,1-Dichloroethane
64	96-24-2	3-Chloro-1,2-propanediol
65	75-09-2	Methylene chloride
66	10318-26-0	Dibromodulcitol
67	79-43-6	Dichloroacetic acid
68	542-56-3	Isobutyl nitrite
69	79-06-1	Acrylamide
70	14484-47-0	Deflazacort
71	50-02-2	Dexamethazone
72	50-23-7	Hydrocortisone
73	78-59-1	Isophorone
74	123-33-1	Maleic hydrazide
75	50-24-8	Prednisolone
76	37076-68-9	Tegafur
77	76-25-5	Triamcinolone acetonide
78	66-22-8	Uracil
79	34661-75-1	Urapidil

No.	CASRN	Chemical Name
80	518-75-2	Citrinin
81	51-21-8	5-Fluorouracil
82	75-07-0	Acetaldehyde
83	100-52-7	Benzaldehyde
84	98-01-1	Furfural
85	129-43-1	1-Hydroxyanthraquinone
86	129-15-7	2-Methyl-1-nitroanthraquinone
87	117-10-2	Danthron
88	81-54-9	Purpurin
89	57-14-7	Dimethyl hydrazine (DMH)
90	34176-52-8	2-Hydrazino-4-phenylthiazole
91	122-66-7	Hydrazobenzene
92	54-85-3	Isoniazid
93	6294-89-9	Methyl carbazate
94	671-16-9	Procarbazine
95	32852-21-4	Formic acid 2-(4-methyl-2-thiazolyl)hydrazide
96	2411-74-7	2-Furaldehyde semicarbazone
97	1156-19-0	Tolazamide
98	25843-45-2	Azoxymethane
99	622-78-6	Benzyl isothiocyanate
100	2257-09-2	Phenethyl isothiocyanate
101	10473-70-8	1-(4-Chlorophenyl)-1-phenyl-2-propynyl carbamate
102	598-55-0	Methyl carbamate
103	51-79-6	Urethane
104	1212-29-9	N,N'-Dicyclohexylthiourea
105	96-45-7	Ethylene thiourea (ETU)
106	13752-51-7	Morpholine, 4-[(4-morpholinylthio)thioxomethyl]-
107	97-77-8	Tetraethylthiuram disulfide
108	137-26-8	Tetramethylthiouram disulfide
109	62-55-5	Thioacetamide
110	62-56-6	Thiourea
111	2489-77-2	Trimethylthiourea
112	105-55-5	N,N'-Diethylthiourea
113	50-32-8	Benzo(a)pyrene
114	56-49-5	3-Methylcholanthrene
115	128-66-5	C.I Vat yellow 4
116	244-63-3	Norharman
117	115-28-6	Chlorendic acid
118	143-50-0	Chlordecone (kepone)
119	39801-14-4	Mirex, photo-
120	2385-85-5	Mirex

No.	CASRN	Chemical Name
121	760-56-5	1-Allyl-1-nitrosourea
122	10589-74-9	1-Amyl-1-nitrosourea
123	16338-97-9	Diallylnitrosamine
124	56654-52-5	1,3-Dibutyl-1-nitrosourea
125	3276-41-3	3,6-Dihydro-2-nitroso-2H-1,2-oxazine
126	3851-16-9	N,N'-Dimethyl-N,N'-dinitrosophthalamide
127	55557-00-1	Dinitrosohomopiperazine
128	38434-77-4	Ethylnitrosocyanamide
129	14026-03-0	R(-)-2-Methyl-N-nitrosopiperidine (S(+)-2-Methyl-N-nitrosopiperidine)
130	16813-36-8	1-Nitroso-5,6-dihydrouracil
131	55090-44-3	N-Nitroso-N-methyl-N-dodecylamine
132	684-93-5	N-Nitroso-N-methylurea
133	55556-92-8	Nitroso-1,2,3,6-tetrahydropyridine
134	51542-33-7	N-Nitrosobenzthiazuron
135	53609-64-6	N-Nitrosobis(2-hydroxypropyl)amine
136	60599-38-4	N-Nitrosobis(2-oxopropyl)amine
137	924-16-3	Nitrosodibutylamine
138	1116-54-7	N-Nitrosodiethanolamine
139	55-18-5	N-Nitrosodiethylamine
140	62-75-9	N-Nitrosodimethylamine
141	86-30-6	N-Nitrosodiphenylamine
142	621-64-7	N-Nitrosodipropylamine
143	17608-59-2	N-Nitrosoephedrine
144	10595-95-6	Nitrosoethylmethylamine
145	614-95-9	Nitrosoethylurethane
146	30310-80-6	Nitrosohydroxyproline
147	26921-68-6	N-Nitrosomethyl-(2-hydroxyethyl) amine
148	614-00-6	Nitrosomethylaniline
149	59-89-2	N-Nitrosomorpholine
150	4515-18-8	Nitrosopipecolic acid
151	930-55-2	N-Nitrosopyrrolidine
152	816-57-9	N-Propyl-N-nitrosourea
153	18883-66-4	Streptozotocin
154	40548-68-3	Tetrahydro-2-nitroso-2H-1,2-oxazine
155	3817-11-6	n-Butyl-N-(4-hydroxybutyl)nitrosamine
156	869-01-2	N-n-Butyl-N-nitrosourea
157	13256-06-9	Dipentylnitrosamine
158	13743-07-2	1-(2-Hydroxyethyl)-1-nitrosourea
159	760-60-1	N-Nitroso-N-isobutylurea
160	13256-11-6	Nitroso-N-methyl-N-(2-phenyl)ethylamine
161	1133-64-8	Nitrosoanabasine

No.	CASRN	Chemical Name
162	625-89-8	N-Nitrosobis(2,2,2-trifluoroethyl) amine
163	42579-28-2	1-Nitrosohydantoin
164	5632-47-3	N-Nitrosopiperazine
165	100-75-4	N-Nitrosopiperidine
166	7519-36-0	Nitrosoproline
167	26541-51-5	N-Nitrosothiomorpholine
168	7227-91-0	1-Phenyl-3,3-dimethyltriazene
169	4164-28-7	Dimethylnitramine
170	598-57-2	Methylnitramine
171	108-05-4	Vinyl acetate
172	611-23-4	o-Nitrosotoluene
173	3688-53-7	AF-2
174	88-73-3	2-Chloronitrobenzene
175	100-00-5	4-Chloronitrobenzene
176	551-92-8	1,2-Dimethyl-5-nitroimidazole
177	606-20-2	2,6-Dinitrotoluene
178	298-00-0	Methyl parathion
179	139-94-6	Nithiazide
180	92-55-7	5-Nitro-2-furanmethanediol diacetate
181	91-23-6	o-Nitroanisole
182	98-95-3	Nitrobenzene
183	1836-75-5	Nitrofen
184	86-57-7	1-Nitronaphthalene
185	607-35-2	8-Nitroquinoline
186	56-38-2	Parathion
187	99-35-4	1,3,5-Trinitrobenzene
188	97-00-7	Dinitrochlorobenzene
189	443-48-1	Metronidazole
190	62-23-7	p-Nitrobenzoic acid
191	613-50-3	6-Nitroquinoline
192	91-76-9	1,3,5-Triazine-2,4-diamine, 6-phenyl-
193	108-78-1	Melamine
194	396-01-0	Triamterene
195	59-05-2	Methotrexate
196	303-34-4	Lasiocarpine
197	22571-95-5	Symphytine
198	315-22-0	Monocrotaline
199	97-53-0	Eugenol
200	52214-84-3	Ciprofibrate
201	77-92-9	1,2,3-Propanetricarboxylic acid, 2-hydroxy-
202	104-76-7	2-Ethylhexanol

No.	CASRN	Chemical Name
203	25812-30-0	Gemfibrozil
204	78-42-2	Tris(2-ethylhexyl)phosphate
205	75330-75-5	Lovastatin
206	131-17-9	Diallyl phthalate
207	85-68-7	Butyl benzyl phthalate
208	87-68-3	Hexachloro-1,3-butadiene
209	127-18-4	Tetrachloroethylene
210	116-14-3	Tetrafluoroethylene
211	79-01-6	Trichloroethylene
212	1825-21-4	Pentachloroanisole
213	476-66-4	Ellagic acid
214	90-43-7	o-Phenylphenol
215	51481-61-9	Cimetidine
216	86315-52-8	Isomazole
217	50-44-2	6-Mercaptopurine
218	58-55-9	Theophylline
219	148-79-8	Thiabendazole
220	73590-58-6	Omeprazole
221	58-93-5	Hydrochlorothiazide
222	54-31-9	Furosemide
223	94-58-6	Dihydrosafrole
224	120-62-7	Piperonyl sulfoxide
225	533-31-3	Sesamol
226	56-23-5	Carbon tetrachloride
227	67-72-1	Hexachloroethane
228	72-43-5	Methoxychlor
229	76-03-9	Trichloroacetic acid
230	51-52-5	6-Propyl-2-thiouracil
231	30516-87-1	3'-Azido-3'-deoxythymidine (AIDS)
232	141-90-2	Thiouracil
233	477-30-5	Colcemid
234	123-73-9	Crotonaldehyde
235	2475-45-8	C.I. Disperse blue 1
236	81-49-2	1-Amino-2,4-dibromoanthraquinone
237	117-79-3	2-Aminoanthraquinone
238	82-28-0	1-Amino-2-methylanthraquinone
239	79-19-6	Thiosemicarbazide
240	142-46-1	2,5-Dithiobiurea
241	13010-08-7	N-Butyl-N'-nitro-N-nitrosoguanidine
242	59-87-0	Nitrofurazone
243	2302-84-3	1-Formyl-3-thiosemicarbazide

No.	CASRN	Chemical Name
244	3570-75-0	Formic acid 2-[4-(5-nitro-2-furyl)-2-thiazolyl]hydrazide
245	555-84-0	1-[(5-Nitrofurfurylidene)amino]-2-imidazolidinone
246	91-93-0	3,3'-Dimethoxybenzidine-4,4'-diisocyanate
247	103-85-5	1-Phenyl-2-thiourea
248	5522-43-0	1-Nitropyrene
249	53-95-2	N-Hydroxy-2-acetylaminofluorene
250	3096-50-2	N-(9-Oxo-2-fluorenyl)acetamide
251	607-57-8	2-Nitrofluorene
252	363-17-7	N-(2-Fluorenyl)-2,2,2-trifluoroacetamide
253	28314-03-6	1-Acetylaminofluorene
254	53-96-3	2-Acetylaminofluorene
255	28322-02-3	4-Acetylaminofluorene
256	67730-10-3	Glu-P-2
257	76180-96-6	IQ
258	943-41-9	N-Nitroso-N-methyl-4-nitroaniline
259	5461-85-8	N-Isobutyl-N'-nitro-N-nitrosoguanidine
260	13010-10-1	N-Pentyl-N'-nitro-N-nitrosoguanidine
261	99-80-9	N-Methyl-N,4-dinitrosoaniline
262	70-25-7	1-Methyl-3-nitro-1-nitroso-guanidine
263	13010-07-6	N-Propyl-N'-nitro-N-nitrosoguanidine
264	34627-78-6	1'-Acetoxysafrole
265	94-52-0	6-Nitrobenzimidazole
266	121-88-0	2-Amino-5-nitrophenol
267	5307-14-2	2-Nitro-p-phenylenediamine
268	2425-85-6	C.I. Pigment red 3
269	712-68-5	2-Amino-5-(5-nitro-2-furyl)-1,3,4-thiadiazole
270	99-56-9	4-Nitro-o-phenylenediamine
271	99-55-8	5-Nitro-o-toluidine
272	99-57-0	2-Amino-4-nitrophenol
273	119-34-6	4-Amino-2-nitrophenol
274	1777-84-0	3-Nitro-p-acetophenetide
275	6471-49-4	C.I. Pigment red 23
276	121-66-4	2-Amino-5-nitrothiazole
277	446-86-6	Azathioprine
278	1582-09-8	Trifluralin, technical grade
279	531-82-8	N-[4-(5-Nitro-2-furyl)-2-thiazolyl]acetamide
280	619-17-0	4-Nitroanthranilic acid
281	33229-34-4	HC blue 2
282	15721-02-5	2,2',5,5'-Tetrachlorobenzidine
283	97-56-3	o-Aminoazotoluene
284	58-14-0	Pyrimethamine

No.	CASRN	Chemical Name
285	80-08-0	4,4'-Sulfonyldianiline (Dapsone)
286	95-80-7	2,4-Diaminotoluene (2,4-toluene diamine)
287	91-94-1	3,3'-Dichlorobenzidine
288	106-50-3	1,4-Benzenediamine
289	133-90-4	Chloramben
290	101-14-4	4,4'-Methylenebis(2-chloroaniline)
291	2243-62-1	1,5-Naphthalenediamine
292	101-80-4	4,4'-Oxydianiline
293	5131-60-2	4-Chloro-m-phenylenediamine
294	95-74-9	3-Chloro-p-toluidine
295	95-79-4	5-Chloro-o-toluidine
296	838-88-0	4,4'-Methylene-bis(2-methylaniline)
297	92-87-5	Benzidine
298	102-50-1	m-Cresidine
299	120-71-8	p-Cresidine
300	609-20-1	2,6-Dichloro-p-phenylenediamine
301	62-53-3	Aniline
302	101-79-1	4-Chloro-4'-aminodiphenylether
303	137-17-7	2,4,5-Trimethylaniline
304	91-59-8	2-Naphthylamine
305	106-47-8	p-Chloroaniline
306	1912-24-9	Atrazine
307	60-11-7	4-Dimethylaminoazobenzene
308	55-80-1	3'-Methyl-4-dimethylaminoazobenzene
309	101-61-1	4,4'-Methylenebis(N,N-dimethyl)benzenamine
310	2784-94-3	HC blue 1
311	121-69-7	N,N-Dimethylaniline
312	90-94-8	Michler's ketone
313	2832-40-8	C.I. Disperse yellow 3
314	398-32-3	N-4-(4'-Fluorobiphenyl)acetamide
315	4463-22-3	3-Hydroxy-4-acetylaminobiphenyl
316	62-44-2	Phenacetin
317	6673-35-4	Practolol
318	77-46-3	4,4'-Sulfonylbisacetanilide
319	18699-02-0	4-Acetylaminophenylacetic acid
320	103-33-3	Azobenzene
321	842-07-9	C.I Solvent yellow 14
322	599-79-1	Salicylazosulfapyridine
323	924-42-5	N-Methylolacrylamide
324	22131-79-9	Alclofenac
325	101-05-3	Anilazine

No.	CASRN	Chemical Name
326	37087-94-8	2-Chloro-5-(3,5-dimethylpiperidinosulphonyl)benzoic acid
327	2698-41-1	o-Chlorobenzalmalononitrile (CS)
328	108-90-7	Chlorobenzene
329	94-20-2	Chlorpropamide
330	106-46-7	1,4-Dichlorobenzene (p-dichlorobenzene)
331	53-86-1	Indomethacin
332	2227-13-6	p-Chlorophenyl-2,4,5-trichlorophenyl sulfide
333	72-55-9	p,p'-Dichlorodiphenyl dichloroethylene
335	115-32-2	Dichlorodiphenyltrichloroethane (DDT)
336	94-59-7	Safrole
337	95-06-7	Sulfallate
338	103-23-1	Di(2-ethylhexyl)adipate
339	133-07-3	N-(Trichloromethylthio)phthalimide
340	23255-69-8	Fusarenon-X
341	765-34-4	Glycidaldehyde
342	106-89-8	Epichlorhydrin
343	76-01-7	Pentachloroethane
344	56980-93-9	Celiprolol
345	101-21-3	Isopropyl-N-(3-chlorophenyl) carbamate
346	135-88-6	N-Phenyl-2-naphthylamine
347	74-31-7	N,N'-Diphenyl-p-phenylenediamine
348	622-51-5	p-Tolylurea
349	5979-28-2	C.I. pigment yellow 16
350	968-81-0	Acetohexamide
351	79-10-7	Acrylic acid
352	107-18-6	Allyl alcohol
353	60-32-2	6-Aminocaproic acid
354	60142-96-3	1-(Aminomethyl)cyclohexaneacetic acid
355	57-43-2	Amobarbital
356	50-81-7	L-Ascorbic acid
357	22839-47-0	Aspartame
358	51-55-8	Atropine
359	71-43-2	Benzene
360	271-89-6	Benzofuran
361	120-32-1	o-Benzyl-p-chlorophenol
362	110-97-4	Diisopropanolamine
363	96-48-0	Gamma-butyrolactone
364	58-08-2	Caffeine
365	105-60-2	Caprolactam
366	7235-40-7	beta-Carotene
367	50892-23-4	(4-Chloro-6-(2,3-xylidino)-2-pyrimidinylthio) acetic acid (WY-14643)

No.	CASRN	Chemical Name
368	22494-47-9	Clobuzarit
369	76-57-3	Codeine
370	31698-14-3	Cyclocytidine
371	1192-28-5	Cyclopentanone oxime
372	53-43-0	Dehydroepiandrosterone
373	333-41-5	Diazinon
374	1717-00-6	Ethane, 1,1-dichloro-1-fluoro-
375	2921-88-2	Chlorpyrifos (Dursban)
376	111-46-6	Diethylene glycol
377	56-53-1	Diethylstilbestrol
378	60-51-5	Dimethoate
379	120-61-6	Dimethyl terephthalate
380	127-19-5	N,N-Dimethylacetamide
381	57-41-0	5,5-Diphenylhydantoin (phenytoin)
382	63-84-3	dl-Dopa
383	2629-59-6	S-Ethyl-l-cysteine
384	100-41-4	Ethylbenzene
385	41340-25-4	Etodolac
386	55-38-9	Fenthion
387	118-74-1	hexachlorobenzene
388	319-84-6	alpha-1,2,3,4,5,6-Hexachlorocyclohexane
389	77-47-4	Hexachlorocyclopentadiene (HCCPD)
390	70-30-4	Hexachlorophene
391	100-97-0	Urotropine
392	136-77-6	4-Hexylresorcinol
393	15687-27-1	Ibuprofen
394	5989-27-5	D-Limonene
395	1634-78-2	Malaoxon
396	89-78-1	dl-Menthol
397	67-98-1	MER-25
398	149-30-4	2-Mercaptobenzothiazole
399	493-78-7	Methaphenilene
400	150-76-5	Hydroquinone monomethyl ether
401	1634-04-4	Methyl-t-butyl ether
402	872-50-4	N-Methyl-2-pyrrolidone
403	98-85-1	alpha-Methylbenzyl alcohol
404	452-86-8	p-Methylcatechol
405	119-47-1	Phenol, 2,2'-methylenebis[6-(1,1-dimethylethyl)-4-methyl-
406	91-62-3	6-Methylquinoline
407	54-11-5	Nicotine
408	139-13-9	Nitrilotriacetic acid (NTA)

No.	CASRN	Chemical Name
409	600-24-8	2-Nitrobutane
410	75-52-5	Nitromethane
411	64224-21-1	Oltipraz
412	23135-22-0	Oxamyl
413	149-29-1	Patulin
414	108-95-2	Phenol
415	77-09-8	Phenolphthalein
416	92-13-7	Pilocarpine
417	110-85-0	Piperazine
418	110-89-4	Piperidine
419	57-66-9	Probenecid
420	121-79-9	Propyl gallate
421	115-07-1	Propylene
422	57-55-6	1,2-Propylene glycol
423	99-50-3	Protocatechuic acid
424	98-96-4	Pyrazinamide
425	108-46-3	Resorcinol
426	127-47-9	Retinol acetate
427	79-81-2	All-trans-retinyl palmitate
428	81-07-2	Saccharin
429	108-30-5	Succinic anhydride
430	107-35-7	L-Taurine
431	732-26-3	Phenol, 2,4,6-tris(1,1-dimethylethyl)-
432	2438-88-2	2,3,5,6-Tetrachloro-4-nitroanisole
433	109-99-9	Ethane, 1,1,1,2-tetrafluoro-
434	91-79-2	Thenyldiamine
435	96-69-5	4,4-Thiobis(6-tert-butyl-m-cresol)
436	64-77-7	Tolbutamide
437	88-19-7	o-Toluenesulfonamide
438	76-13-1	1,1,2-Trichloro-1,2,2-trifluoroethane, technical grade
439	71-55-6	1,1,1-Trichloroethane, technical grade
440	75-69-4	Trichlorofluoromethane
441	88-06-2	2,4,6-Trichlorophenol
442	112-27-6	Triethylene glycol
443	127-48-0	Trimethadione
444	458-37-7	Turmeric (98% curcurmin)
445	57-13-6	Urea
446	88-12-0	2-Pyrrolidinone, 1-ethenyl-
447	127-06-0	Acetoxime
448	616-91-1	N-acetylcysteine
449	2835-39-4	Allyl isovalerate

No.	CASRN	Chemical Name
450	2432-99-7	11-Aminoundecanoic acid
451	4180-23-8	Benzene, 1-methoxy-4-(1E)-1-propenyl-
452	65-85-0	Benzoic acid
453	331-39-5	3,4-Dihydroxycinnamic acid
454	853-23-6	Dehydroepiandrosterone acetate
455	95-50-1	1,2-Dichlorobenzene (o-dichlorobenzene)
456	94-75-7	2,4-Dichlorophenoxyacetic acid
457	685-91-6	Diethylacetamide
458	62488-57-7	5,6-Dihydro-5-azacytidine
459	13265-60-6	O,O-Dimethyl S-2(acetylamino)ethyl dithiophosphate, technical grade
460	13073-35-3	Ethionine (DL-ethionine)
461	64-17-5	Ethanol
462	111-68-2	Heptylamine
463	148-24-3	8-Hydroxyquinoline
464	115-11-7	Isobutene
465	121-75-5	Malathion
466	531-06-6	Methafurylene
467	112-63-0	Methyl linoleate, native
468	578-76-7	7-Methylguanine
469	95-71-6	Methylhydroquinone
470	79-24-3	Nitroethane
471	79-46-9	2-Nitropropane
472	50-06-6	Phenobarbital
473	89-25-8	1-Phenyl-3-methyl-5-pyrazolone
474	1918-02-1	Picloram, technical grade
475	105-11-3	p-Benzoquinone dioxime
476	23031-25-6	Terbutaline
477	1972-08-3	1-trans-delta-9-Tetrahydrocannabinol
478	538-23-8	Tricaprylin
479	95-63-6	Benzene, 1,2,4-trimethyl-
480	75-38-7	Vinylidene fluoride

S3 Distribution of prediction combinations

Prediction: 0 ⇒ Non-carcinogen 1 ⇒ Carcinogen

Table S3: Prediction combination table with number of chemicals with the prediction combination for Dataset 1. The dataset consists of 332 chemicals with a carcinogen to non-carcinogen ratio of 114:218.

Combination Number	Toxtree	Lazar	Danish QSAR	OECD Toolbox	Number of chemicals
s_1	0	0	0	0	110
s_2	0	0	0	1	0
s_3	0	0	1	0	27
s_4	0	0	1	1	0
s_5	0	1	0	0	30
s_6	0	1	0	1	1
s_7	0	1	1	0	29
s_8	0	1	0	1	1
s_9	0	1	1	1	2
s_{10}	1	0	0	0	9
s_{11}	1	0	0	1	0
s_{12}	1	0	1	0	35
s_{13}	1	0	1	1	0
s_{14}	1	1	0	0	14
s_{15}	1	1	0	1	0
s_{16}	1	1	1	1	57

Table S4: Prediction combination table with number of chemicals with the prediction combination for Dataset 2. The dataset consists of 480 chemicals with a carcinogen to non-carcinogen ratio of 258:222.

Combination Number	Toxtree	Lazar	Danish QSAR	OECD Toolbox	Number of chemicals
s_1	0	0	0	0	40
s_2	0	0	0	1	6
s_3	0	0	1	0	48
s_4	0	0	1	1	5
s_5	0	1	0	0	8
s_6	0	1	0	1	1
s_7	0	1	1	0	28
s_8	0	1	0	1	3
s_9	0	1	1	1	1
s_{10}	1	0	0	0	29
s_{11}	1	0	0	1	2
s_{12}	1	0	1	0	70
s_{13}	1	0	1	1	1
s_{14}	1	1	0	0	14
s_{15}	1	1	0	1	5
s_{16}	1	1	1	1	219
