# Supplementary material for: An ensemble model of QSAR tools for regulatory risk assessment
Source: J Cheminform. 2016 Sep 22;8:48. doi: 10.1186/s13321-016-0164-0 (PMC5034616; doi:10.1186/s13321-016-0164-0)
Supplement: Supplementary file 2 — 10.1186/s13321-016-0164-0 Matlab generated code file and is self-explanatory. [file 13321_2016_164_MOESM2_ESM.pdf]

Table of Contents

.... 1
Develop the Model 1
Calculate prediction metrics 3
Generate ROC Plot 5


```
% BayesEnsembleModel_LOO.m
% Code to develop a Bayesian ensemble model to combine predictions
 from four independent QSAR
% tools. The final Bayesian prediction is based on a probabilistic
 cut-off.
%
% Author: Prachi Pradeep
```

Develop the Model

```
% Read the input data file (each dataset has its own input file)
% File format: Col. 1: Experimental Data, Col. 2: Toxtree pred, Col.
 3: Lazar pred, Col. 4: Danish QSAR pred, Col 5.: OECD Toolbox pred.
Input_data = xlsread('Inhalation.xls');
%Input_data = xlsread('CPDB.xlsx');

%Individual predictions for each tool
Experimental = Input_data(:,1);
Toxtree = Input_data(:,2);
Lazar = Input_data(:,3);
Danish = Input_data(:,4);
OECD = Input_data(:,5);

Tools = [Toxtree,Lazar,Danish,OECD];
[n,c] = size(Tools);

% Each of the 4 tool prediction is considered a vote (0, 1, 2, 3 or
 4).
% Each combination is a result of permutation of these 4 votes.
% The combination of votes can result in 4^2(= 16) prediction
 combinations.

% Calculate prediction combination and sum of votes for each chemical.
pred_comb = 8*Toxtree+4*Lazar+2*Danish+1*OECD;
pred_votes = sum([Toxtree,Lazar,Danish,OECD],2);
Data = [Toxtree, Lazar, Danish, OECD, Experimental, pred_comb,
 pred_votes];

% Each of these combinations is assigned a bin and a corresponding
 vote count.
bins = [0,1,2,3,4,5,6,7,8,9,10,11,12,13,14,15]'; % Prediction
 combinations
```

```
votes = [0,1,1,2,1,2,2,3,1,2,2,3,2,3,3,4]'; % Corresponding number of
 votes
no_bins = length(bins);

probability_cutoffs = (0:.1:1);
len = length(probability_cutoffs); % Number of cut-off points
Model = zeros(n,len); % Model predictions for each cut-off

% Leave One Out cross validation - Create n training sets of n-1 data
 points
% each and predict the value for the left out (nth) data point
for i = 1:n
    training = Data;
    training(i,:) = []; %Delete the ith row to be used as test data

    total = zeros(no_bins,1); %Total chemicals in each prediction
 combination
    active_count = zeros(no_bins,1); %Total actives (carcinogens) in
 each combination
    prob = zeros(no_bins,1); % Probability of actives (carcinogens) in
 each combination

    %Calculate probability of actives (carcinogens) in each
 combination
    for j = 1:no_bins
            total(j) = length(find(training(:,6) == j-1)); %Col 6:
 Prediction combination
            active_count(j) = length(find(training(:,6) == j-1
 & training(:,5) == 1)); %Col 6: Prediction combination, Col 5:
 Experimental data
            if total(j) == 0
                total(j) = 100000; %To avoid division by zero
            end
            prob(j) = active_count(j)/total(j); %Prior probability of
 actives for each prediction combination
    end
    prob_matrix = [bins, votes, prob]; %Prediction combination,
 associated votes and calculated priors

    Test_combination = Data(i,6); % Prediction combination for the
 Test chemical in the LOO analysis
    Test_prob_active =
 prob_matrix(find(prob_matrix(:,1)==Test_combination),3); %Posterior
 probability based on the prior for the test chemical

    % Compare the probability with the cut-off to arrive at a decision
    for k = 1:len
        if Test_prob_active >= probability_cutoffs(k)
            Model(i,k) = 1; % Label active
        else
            Model(i,k) = 0; % Label inactive
        end
    end
end
```

Calculate prediction metrics

```
Total_pos = size(find(Experimental == 1),1); %Total actives in the
 dataset
Total_neg = size(find(Experimental == 0),1); %Total inactives in the
 dataset

% Calculate TP, TN, FP, FN for each of the tools and the Bayes
 ensemble model
Toxtree_TP = 0;
Lazar_TP = 0;
Danish_TP = 0;
OECD_TP = 0;
Model_TP = zeros(1,k);

Toxtree_TN = 0;
Lazar_TN = 0;
Danish_TN = 0;
OECD_TN = 0;
Model_TN = zeros(1,k);

for i = 1:n
    if Experimental(i) == 1
        if Toxtree(i) == Experimental(i)
            Toxtree_TP = Toxtree_TP+1;
        end
        if Lazar(i) == Experimental(i)
            Lazar_TP = Lazar_TP+1;
        end
        if Danish(i) == Experimental(i)
            Danish_TP = Danish_TP+1;
        end
        if OECD(i) == Experimental(i)
            OECD_TP=OECD_TP+1;
        end
        for k = 1:len
             if Model(i,k) == Experimental(i)
                Model_TP(k) = Model_TP(k)+1;
            end
        end
    end
    if Experimental(i) == 0
        if Toxtree(i) == Experimental(i)
            Toxtree_TN = Toxtree_TN+1;
        end
        if Lazar(i) == Experimental(i)
            Lazar_TN = Lazar_TN+1;
        end
        if Danish(i) == Experimental(i)
            Danish_TN = Danish_TN+1;
        end
        if OECD(i) == Experimental(i)
            OECD_TN = OECD_TN+1;
```

```
            end
            for k = 1:len
                if Model(i,k) == Experimental(i)
                    Model_TN(k) = Model_TN(k)+1;
                end
            end
        end
    end

    % Calculate Accuracy
    acc_Toxtree = 100*(Toxtree_TP + Toxtree_TN)/(Total_pos + Total_neg);
    acc_Lazar = 100*(Lazar_TP + Toxtree_TN)/(Total_pos + Total_neg);
    acc_Danish = 100*(Danish_TP + Toxtree_TN)/(Total_pos + Total_neg);
    acc_OECD = 100*(OECD_TP + Toxtree_TN)/(Total_pos + Total_neg);
    acc_Model = 100*(Model_TP + Model_TN)/(Total_pos + Total_neg);

    Accuracy = [acc_Toxtree, acc_Lazar, acc_Danish, acc_OECD,
     acc_Model(6)]; % acc_Model(6): Predicted value at a probability cut-
    off of 0.5

    % Calculate Sensitivity
    Sense_Toxtree = Toxtree_TP/Total_pos;
    Sense_Lazar = Lazar_TP/Total_pos;
    Sense_Danish = Danish_TP/Total_pos;
    Sense_OECD = OECD_TP/Total_pos;
    Sense_Model = Model_TP/Total_pos;
    Sensitivity = [Sense_Toxtree, Sense_Lazar, Sense_Danish, Sense_OECD,
     Sense_Model(6)]; % Sense_Model(6): Predicted value at a probability
     cut-off of 0.5

    % Calculate Specificity
    Spec_Toxtree = Toxtree_TN/Total_neg;
    Spec_Lazar = Lazar_TN/Total_neg;
    Spec_Danish = Danish_TN/Total_neg;
    Spec_OECD = OECD_TN/Total_neg;
    Spec_Model = Model_TN/Total_neg;
    Specificity = [Spec_Toxtree, Spec_Lazar, Spec_Danish, Spec_OECD,
     Spec_Model(6)]; % Spec_Model(6): Predicted value at a probability
     cut-off of 0.5

    % Calculate PPV and NPV
    Toxtree_Pos = size(find(Toxtree == 1),1);
    Toxtree_Neg = size(find(Toxtree == 0),1);
    Lazar_Pos = size(find(Lazar == 1),1);
    Lazar_Neg = size(find(Lazar == 0),1);
    Danish_Pos = size(find(Danish == 1),1);
    Danish_Neg = size(find(Danish == 0),1);
    OECD_Pos = size(find(OECD == 1),1);
    OECD_Neg = size(find(OECD == 0),1);

    Model_Pos = zeros(1,len);
    Model_Neg = zeros(1,len);
    for i = 1:len
            Model_Pos(i) = size(find(Model(:,i) == 1),1);
```

```
        Model_Neg(i) = size(find(Model(:,i) == 0),1);
end

PPV_Toxtree = Toxtree_TP*100/Toxtree_Pos;
PPV_Lazar = Lazar_TP*100/Lazar_Pos;
PPV_Danish = Danish_TP*100/Danish_Pos;
PPV_OECD = OECD_TP*100/OECD_Pos;
PPV_Model = Model_TP*100./Model_Pos;

NPV_Toxtree = Toxtree_TN*100/Toxtree_Neg;
NPV_Lazar = Lazar_TN*100/Lazar_Neg;
NPV_Danish = Danish_TN*100/Danish_Neg;
NPV_OECD = OECD_TN*100/OECD_Neg;
NPV_Model = Model_TN*100./Model_Neg;

PPV = [PPV_Toxtree, PPV_Lazar, PPV_Danish, PPV_OECD, PPV_Model(6),
 PPV_Model(7), PPV_Model(5)];
NPV = [NPV_Toxtree, NPV_Lazar, NPV_Danish, NPV_OECD, NPV_Model(6),
 NPV_Model(7), NPV_Model(5)];
```

Generate ROC Plot

```
hFig = figure(1);
set(gcf,'Color',[1,1,1])
set(hFig, 'Position', [80 80 1000 600])
plot(1-Specificity,
 Sensitivity,'o','MarkerFaceColor','b','MarkerEdgeColor','k','MarkerSize',8)
label1 = cellstr(char('Toxtree'));
label2 = cellstr(char('Lazar','Danish QSAR'));
label3 = cellstr(char('OECD Toolbox','Bayes'));
text(1-Specificity(1), Sensitivity(1),
 label1, 'VerticalAlignment','bottom','HorizontalAlignment','left','FontSize',12);
text(1-Specificity(2:3), Sensitivity(2:3),
 label2, 'VerticalAlignment','bottom','HorizontalAlignment','left','FontSize',12);
text(1-Specificity(4:5), Sensitivity(4:5),
 label3, 'VerticalAlignment','top','HorizontalAlignment','left','FontSize',12);
title('ROC Curve - Air Toxins Dataset (315 compounds)');
%title('ROC Curve - Gold Carcingenic Potency Database (480
 compounds)');
hold on
plot((0:1),(0:1),'k')
plot(1-Spec_Model,Sense_Model,'--rs','LineWidth',2,...
                'MarkerEdgeColor','k',...
                'MarkerFaceColor','r',...
                'MarkerSize',10)
plot(1-Spec_Model(6),Sense_Model(6),'--rs','LineWidth',2,...
                'MarkerEdgeColor','k',...
                'MarkerFaceColor','g',...
                'MarkerSize',10)
legend('QSAR Tools','Random Classifier','ROC curve for
 Bayes Ensemble Model', 'Bayes Ensemble Model (Cut-
off=0.5)', 'Location', 'Southeast')
hold off
```

```
ylabel('Sensitivity');
xlabel('1 - Specificity');
```

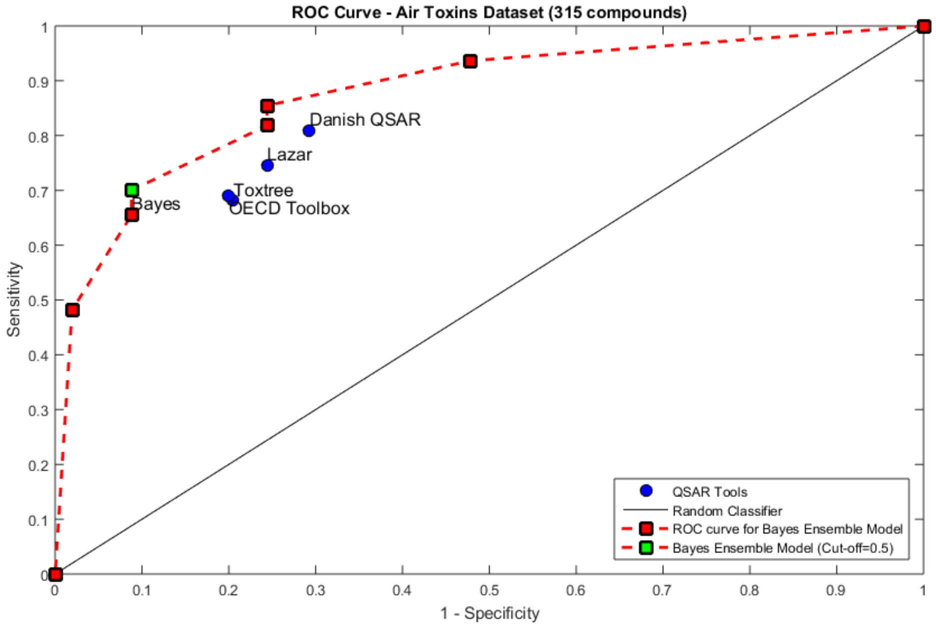

Published with MATLAB® R2015a
